# Short-Snouted Toothless Ichthyosaur from China Suggests Late Triassic Diversification of Suction Feeding Ichthyosaurs

**DOI:** 10.1371/journal.pone.0019480

**Published:** 2011-05-23

**Authors:** P. Martin Sander, Xiaohong Chen, Long Cheng, Xiaofeng Wang

**Affiliations:** 1 Steinmann Institute, Division of Palaeontology, University of Bonn, Bonn, Germany; 2 Wuhan Institute of Geology and Mineral Resources (formerly Yichang Institute of Geology and Mineral Resources), Hubei, China; College of the Holy Cross, United States of America

## Abstract

**Background:**

Ichthyosaurs were an important group of Mesozoic marine reptiles and existed from the Early Triassic to the early Late Cretaceous. Despite a great diversity in body shapes and feeding adaptations, all share greatly enlarged eyes, an elongated rostrum with numerous conical teeth, and a streamlined body.

**Methodology/Principal Findings:**

Based on new material from China and the restudy of *Shastasaurus pacificus*, we here reinterpret the classical large-bodied Late Triassic ichthyosaur genus *Shastasaurus* to differ greatly from the standard ichthyosaurian body plan, indicating much greater morphological diversity and range of feeding adaptations in ichthyosaurs than previously recognized. Phylogenetic analysis indicates a monophyletic clade consisting of the giant *Shonisaurus sikanniensis*, *Guanlingsaurus liangae*, and *Shastasaurus pacificus* to which the genus name *Shastasaurus* is applied. *Shastasaurus liangae* comb. nov. is from the Late Triassic (Carnian) Xiaowa Formation of Guizhou Province, southwestern China. The species combines a diminutive head with an entirely toothless and greatly reduced snout. The species also has by far the highest vertebral count among ichthyosaurs (86 presacral vertebrae and >110 caudal vertebrae), a count that is also very high for tetrapods in general. A reduced toothless snout and a diminutive head is also apparently present in the giant *S. sikanniensis* and presumably in *S. pacificus*.

**Conclusions/Significance:**

In analogy to many modern odontocetes, *Shastasaurus* is interpreted as a specialized suction feeder on unshelled cephalopods and fish, suggesting a unique but widespread Late Triassic diversification of toothless, suction-feeding ichthyosaurs. Suction feeding has not been hypothesized for any of the other diverse marine reptiles of the Mesozoic before, but in *Shastasaurus* may be linked to the Late Triassic minimum in atmospheric oxygen.

## Introduction

The Triassic witnessed an unprecedented radiation of marine reptiles, possibly triggered by the Permian/Triassic extinction event which left the marine environment largely devoid of metazoans [1], [2] or by the dramatic decline of atmospheric oxygen level [3], [4] during this period. The most successful group in this radiation were the ichthyosaurs [5]–[9] which appear in the fossil record in the latest Early Triassic and reached their greatest diversity during the Middle and Late Triassic [5]–[8]. Ichthyosaurs were very well adapted to the marine environment as evidenced by their worldwide record in open marine sediments. This invasion of the marine realm was faciliated by live birth which is already seen in the earliest forms [5]–[9]. Ichthyosaurs are characterized by their greatly enlarged eyes, elongated rostrum and numerous conical teeth.

Ichthyosaurs show many evolutionary convergences with modern cetaceans [5], [6], [8], providing important clues to functional versus phylogenetic constraints in whale evolution. As attested to by fossil stomach contents, the elongated and toothed ichthyosaur rostrum was used to capture fish and squid, the diet of most extant odontocetes. Some of these odontocetes, such as the beaked whales (Ziphiidae), some delphinids, pygmy and dwarf sperm whales (*Kogia sima* and *K. breviceps*), and even the sperm whales (*Physeter*), use suction feeding instead of an elongate tooth-bearing rostrum to capture their prey [10], [11], [12]. In fact, “suction feeding is the dominant method of prey capture in aquatic vertebrates” [11, p. 1415] but has not been postulated for Mesozoic marine reptiles with the exception of the giant *Shonisaurus sikanniensis* [13].

Based on new ichthyosaur finds from China, and the reexamination and reinterpretation of material from the western USA, we suggest that in the Late Triassic there was a previously unrecognized global diversification of large suction-feeding ichthyosaurs that probably were the ecological equivalent to the extant suction-feeding odontocetes.

Large-bodied (adult length >7 m) Late Triassic Ichthyosauria include taxa with the familiar elongated rostrum equipped with numerous teeth [5], [6] but also a few forms lacking teeth combined with an abbreviated snout. These include *Shastasaurus liangae* comb. nov. [14] from the early Carnian of China, *Shastasaurus sikanniensis* comb. nov. [13] from the middle Norian of British Columbia, and probably *Shastasaurus pacificus* [15] from the late Carnian of California.

The youngest of the Chinese Triassic ichthyosaur-bearing formations is the early Carnian Xiaowa Formation of the Guanling area, Guizhou Province [16]. The faunistically unique Xiaowa Formation is also known as the Wayao Formation or the Wayo Member of the Falang Formation in the literature [16]. The fossiliferous black shales represent the upper part of the lower member of the Xiaowa Formation [16]. Ichthyosaurs belonging to three different taxa are the most common vertebrate fossils in these black shales, whereas fish fossils are extremely rare [16]. Most common among the ichthyosaur taxa is the small (total length <2 m) *Qianichthyosaurus zhoui*, followed by the moderately rare and larger (total length <6 m) *Guizhouichthyosaurus tangae* (probable junior synonyms of this taxon are *Cymbspondylus asiaticus* and *Pangjiangsaurus epicharis* [16]), and the large (total length <9 m) *Shastasaurus liangae* comb. nov., previously only known from the poorly prepared holotype of *Guanlingsaurus liangae* housed at the Geological Survey of Guizhou Province, Guiyang, People's Republic of China [16]. Field work by staff of the Wuhan Institute of Geology and Mineral Resources (the former Yichang Institute of Geology and Mineral Resources [YIGMR]) resulted in the aquisition of three excellent new specimens of this species. These and *Shastasaurus pacificus* [5], [15] are the focus of our study.

## Materials and Methods

The specimes of *S. liangae* comb. nov. examined first-hand for this study are the following three individuals: YIGMR SPCV03107, a large but incomplete skeleton, YIGMR SPCV03108, a complete but diagenetically flattened skeleton of a juvenile, and YIGMR SPCV03109, a large and complete but not yet fully prepared skeleton preserverd in three dimensions. In the collections of the Museum of Paleontology, University of California at Berkeley, USA (UCMP), the first author also examined the proposed neotype of *Shastasaurus pacificus* (UCMP 9017) [5], a partial skeleton from the Carnian Hosselkus Limestone of Shasta County, California, USA. This find comprises the skull lacking the snout, the cervical and anterior dorsal vertebral column and ribs, and parts of the shoulder girdle and forelimbs [15].

The phylogenetic framework for this study was obtained through two different phylogenetic analyses. One is based on a modified and extended data matrix of Motani [17] and the other on a modified and extended data matrix of Sander [6]. The data matrices were edited with MacClade 4 and analyzed with PAUP 4.0b10. The matrices were modified by adding four new terminal taxa, *Shastasaurus liangae* comb. nov., *Guizhouichthyosaurus tangae* [14], [18], [19], *Shastasaurus sikanniensis* comb. nov. [13], and *Callawaya* [20]. In addition, six new characters were added (Table S1, Table S2, Table S3, Table S4). *Shastasaurus pacificus* was recoded in the matrices based on personal inspection by P.M.S. in 2007 to only include the material from the late Carnian Hosselkuss Limestone of California [5], [15] for reasons explained below. The modified matrix based on Motani [17] has 36 taxa and 111 characters, and that based on Sander [6] has 15 taxa (the Neoichthyosauria being treated as single terminal taxon) and 125 characters. The search mode was heuristic and employed exactly the same settings as in the original analyses. The resulting trees were optimized both under DELTRAN and ACCTRAN character optimization, but only unambiguous character state transformations (Table S5) were used for inferences about character evolution.

## Results

### Systematic Paleontology

Ichthyosauria Blainville, 1835

Merriamosauria Motani, 1999

*Shastasaurus* Merriam, 1895

#### Type species

*Shastasaurus pacificus* Merriam, 1895

#### Included species

*Shastasaurus sikanniensis* comb. nov. [13], *Shastasaurus liangae* comb. nov. [14].

#### Revised diagnosis based on the phylogenetic analysis

Large to gigantic Shastasauridae diagnosed by the following unambiguous and unequivocal synapomorphies (Table S5): abbreviated rostrum and extremely slender lower jaw. Unambiguous but equivocal (i.e. consistency index is <1) synapomorphies are the lack of a parietal ridge and the loss of marginal teeth. Three ambiguous and equivocal synapomorphies are listed in Table S5. *Shastasaurus* differs from all other ichthyosaurs in the reduced snout and lack of teeth. In addition, *Shastasaurus* differs from other basal Merriamosauria except for *Shonisaurus popularis* and *Besanosaurus* in its larger size.

#### Remarks

The character state “loss of marginal teeth” is also unambiguous and unequivocal if *Hupehsuchus*, which is not an ichthyosaur [5]–[8], is deleted from the analysis. While the holotype of the type species *S. pacificus* is incompletely preserved, it shows at least one of the synapomorphies of the genus *Shastasaurus*, the slender lower jaw. Character optimization indicates that both the lack of teeth and the reduced snout must have been present in *S. pacificus* despite them not being preserved.

#### Horizon and localities

Upper Triassic, lower Carnian to middle Norian of southwestern China and western North America (California and British Columbia).

#### *Shastasaurus liangae* comb. nov

#### Synonymy

*Guanlingsaurus liangae* (Yin in [14])

#### Holotype

Geological Survey of Guizhou Province, Guiyang, People's Republic of China specimen GMR 014, a complete skeleton. However, there are doubts about the integrity of the material. The specimen was briefly described and figured by Yin et al. [14] as *Guanlingsaurus liangae* and access is limited [16], [18], [19]. However, autapomorphies of the species such as the very high number of presacral and caudal vertebrae are clearly discernable from the publication of Yin et al. [14].

#### Referred material

YIGMR SPCV03107, YIGMR SPCV03108, and YIGMR SPCV03109.

#### Horizon and locality

Upper Triassic, lower Carnian laminated beds of the lower Xiaowa Formation, Guanling County, Guizhou Provice, southwestern China [14], [16].

#### Revised diagnosis based on the phylogenetic analysis

Large *Shastasaurus* with a very small skull, less than 10% of total length. *S. liangae* comb. nov. is diagnosed by the following unambiguous but equivocal synapomorphies: postorbital triradiate in shape and contiguous shaft of ulna absent. Other characters are that the rostrum is greatly reduced in length, mainly resulting from very short and slender premaxillae and dentaries. The nasals and the angulars of the lower jaw reach the tip of the snout. The jaws are completely toothless. There are 86 presacral and >110 caudal vertebrae, the highest number of any ichthyosaur [5]–[7]. *S. liangae* comb. nov. differs from *Shastasaurus sikanniensis* comb. nov. [13] in the lacrimal having numerous small to medium-sized nutritive foramina, the supratemporal extending well posterior of the parietal, and in a more strongly foreshortened propodium and zeugopodium in both the forelimb and the hindlimb. *S. liangae* comb. nov. differs from *S. sikanniensis* comb. nov. [13] and *S. pacificus* [5], [15] in the lack of a preaxial notch in the radiale. Differs from *S. pacificus* in the relative longer postorbital region and larger upper temporal openings, the long axes of which are nearly parallel in *S. liangae* but enclose an angle of about 60° in *S. pacificus* because of its posteriorly diverging parietals.

### Description of *Shastasaurus liangae* comb. nov

**Figure 1:**
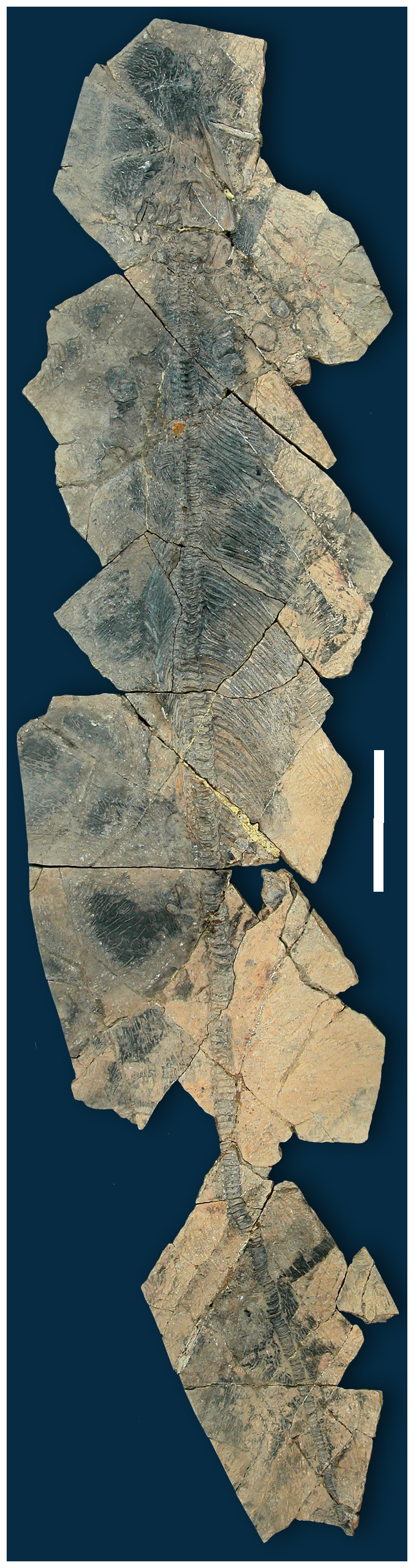
*Shastasaurus liangae* comb. nov. Juvenile individual YGMIR SPCV03108, total length 3.75 m. Scale bar, 50 cm.

**Figure 2:**
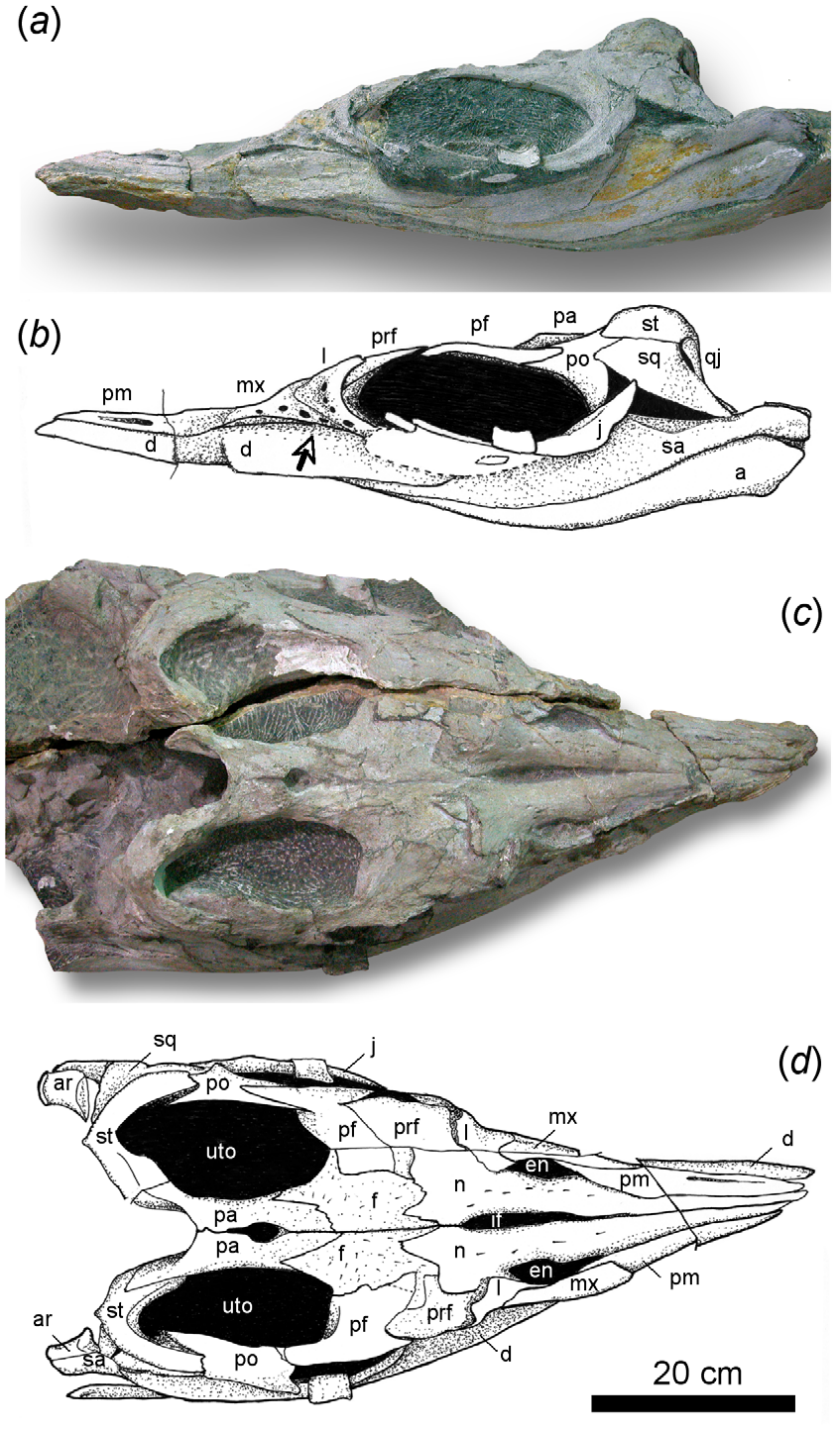
Skull anatomy of *Shastasaurus liangae* comb. nov. Photograph and drawing of skull of YGMIR SPCV03107. (A) in left lateral view. Note the greatly abbreviated rostrum, the complete lack of teeth, the large foramina in the maxillary and lacrimal bones, and the dorsally convex coronoid region of the dentary (arrow). (B) in dorsal view. Note the nasals extending to the tip of the rostrum. Abbreviations: a, angular; ar, articular; d, dentary; en, external nares; f, frontal; if, internasal foramen; j, jugal; l, lacrimal; mx, maxilla; pa, parietal; pf, postfrontal; pm, premaxilla; po, postorbital; prf, prefrontal; qj, quadratojugal; sa, surangular; sq, squamosal; st, supratemporal; uto, upper temporal opening.

**Figure 3:**
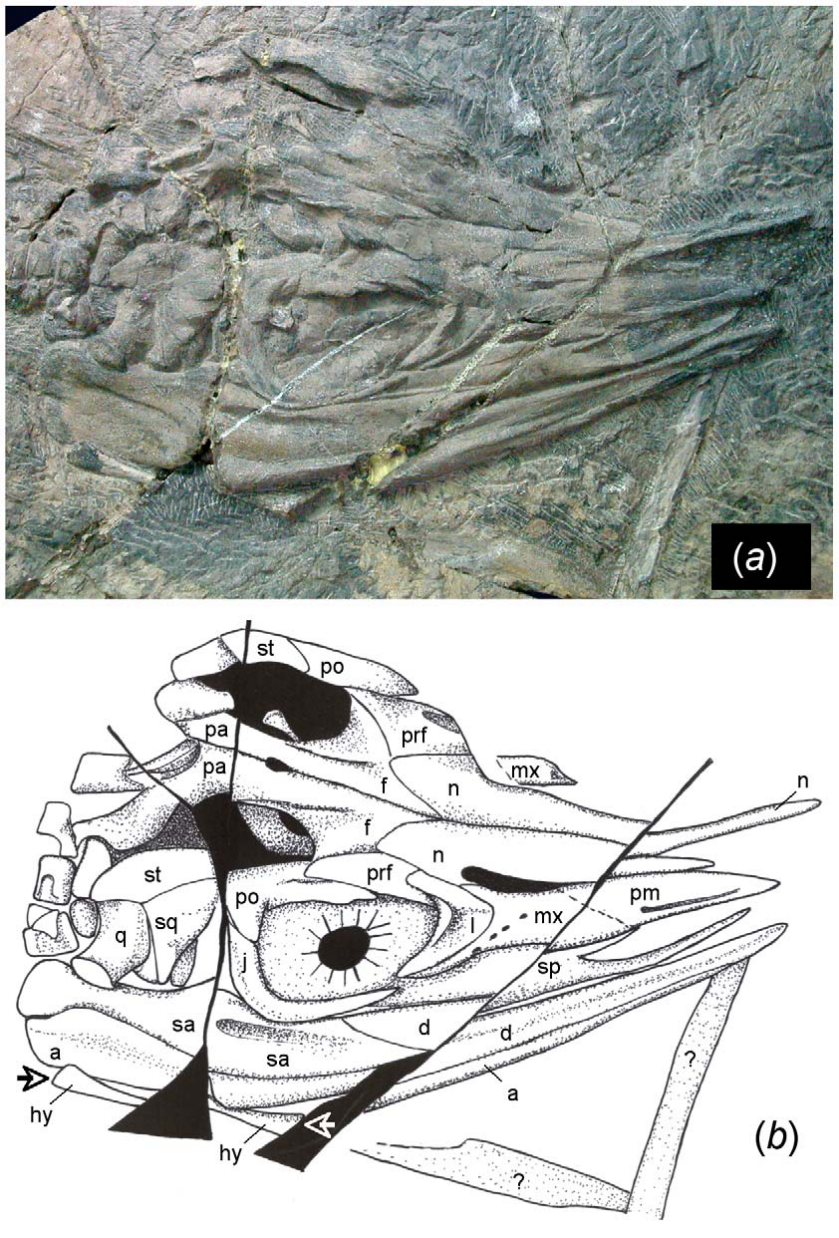
Skull anatomy of *Shastasaurus liangae* comb. nov. Photograph (A) and drawing (B) of the skull of the juvenile specimen YGMIR SPCV03108. Crushing lead to both the dorsal and the right lateral view being exposed. Note the greatly abbreviated rostrum, the complete lack of teeth, and the large foramina in the maxilla. Also note the nasals extending to the tip of the rostrum and the angular almost extending to the tip of the lower jaw. The extent of the left hyoid bone is highlighted by the arrows. Abbreviations: a, angular; d, dentary; f, frontal; hy, hyoid bone: j, jugal; l, lacrimal; mx, maxilla; pa, parietal; pf, postfrontal; pm, premaxilla; po, postorbital; prf, prefrontal; q, quadrate; sa, surangular; sq, squamosal; st, supratemporal.

The largest skeleton of *S. liangae* (YIGMR SPCV03109) is 8.3 m long. This is somewhat longer than the holotype and slightly longer than the three-dimensionally preserved specimen YGMIR SPCV03107 which must have been about 7 m in total length. The juvenile (YIGMR SPCV03108, Fig. 1) is 3.74 m long. Skull length as measured along skull midline is 8.3% of total length in the largest specimen and 9.3% in the juvenile. Skull length is 17.7% of presacral lenght in the juvenile compared to >40% in most other ichthyosaurs [6]. The most striking feature about the skull of *S. liangae* comb. nov. is its very short snout region (Figs. 2, 3). In addition, the snout is completely toothless, as best shown by the juvenile skull because of the partial disarticulation of its jaw bones. There is no evidence for a dental groove in the dentary, premaxilla, and maxilla. All bones contributing to the snout taper rapidly to a point. The premaxilla is dominated by an elongate foramen that enters the bone obliquely in posterior direction. Likewise, the maxilla shows several very large foramina that take up much of the lateral side of the bone and are not seen in any other ichthyosaur. The maxilla is excluded from the external nares by the premaxilla and lacrimal. There is a very large internasal foramen between the external nares. The lacrimal is perforated by numerous small to medium-sized foramina. Uniquely among ichthyosaurs [5], and extremely unusual among sauropsids, the nasal extends anteriorly to the very tip of the snout (Fig. 2C, D). The orbit is evenly oval in outline, and the orbital and postorbital region of the skull are as in other Merriamosauria [5].

The dentaries of the lower jaw are very short and completely edentulous with a smooth dorsal surface. In lateral view, there is a marked dorsal convexity in the coronoid region of the dentary opposite the maxilla, which is ventrally concave. Just as unusual as the nasal extending to tip of the upper jaw is the configuration of the angular that ventrally, together with the splenial, extends to the very tip of the lower jaw, as can be seen in the skull YIGMR SPCV03109 in ventral view and in the juvenile YIGMR SPCV03108 (Fig. 3). The postdentary region of the lower jaw is as in other Merriamosauria [5] and ends in a large retroarticular process. The hyoid bones are only observable in the juvenile and are remarkably long, reaching 31% of the length of the lower jaw.

*Shastasaurus liangae* comb. nov. has by far the highest number of vertebrae of any ichthyosaur [5], [6] with approximately 86 presacrals and over 100 caudal vertebrae (Fig. 1). This is also among the highest numbers in amniotes in general [21]. From the first cervical to the middle dorsal, the vertebrae nearly double in height and width. Compared to other Merriamosauria, the loss of contact of the diapophysis with the neural arch occurs very far back, on the 69th presacral. Despite the very high number of vertebrae, the body shape index [13] of 3.9 is similar to other long-bodied Triassic Merriamosauria [13]. The caudal vertebral column is very straight without a tailbend (Fig. 1).

**Figure 4:**
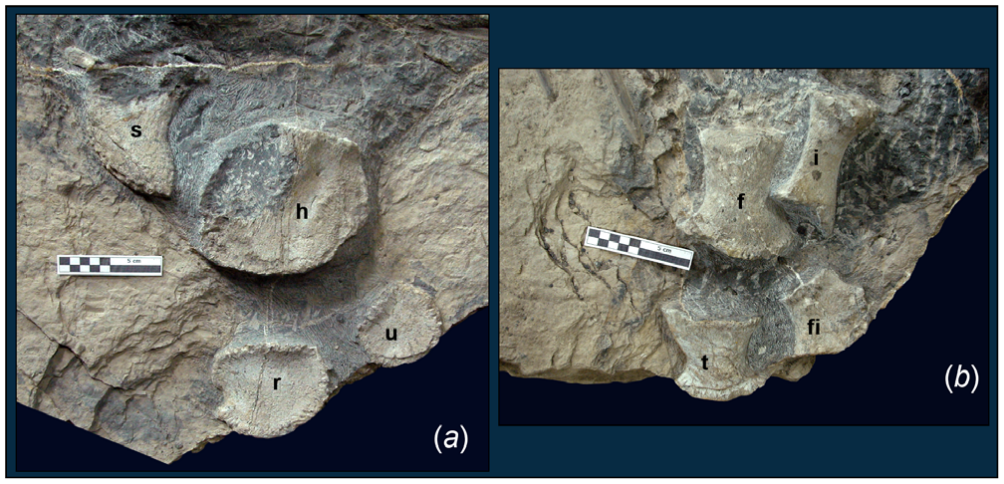
Appendicular skeleton of *Shastasaurus liangae* comb. nov. (A) Pectoral girdle and forelimb elements of YGMIR SPCV03107. (B) Pelvic girdle and hindlimb elements of YGMIR SPCV03107. Abbreviations: f, femur; fi, fibula; h, humerus; i, ischium; r, radius; s, scapula; t, tibia; u, ulna. Scale bars, 10 cm.

The appendicular skeleton is generally similar to that of other basal Merriamosauria, with the proximal bones of the anterior limbs all being disc-shaped, suggesting that they were surrounded by extensive cartilage (Fig. 4A). Distally, the forefins appear to have been incompletely ossified as well, considering that all three new specimens preserve very few fin bones except for humerus, radius, ulna and radiale. This suggests that the distal carpals, metacarpals, and phalanges possibly were not ossified at all. The alternative explanation, that they were lost taphonomically, is inconsistent with the high degree of articulation generally observed in marine reptiles from the Xiaowa Formation [16]. If these elements were ossified, it appears likely that at least one out of three specimens should have preserved some of these bones. The incomplete ossification of the distal forelimbs is also suggested by the illustration of the holotype with only three digits preserved and an apparent phalangeal formula of 1-1-0 [14, plate 9]. The humerus of *S. liangae* is wider than long, and its length is only 1% of the body length. Together with the small zeugopodium of the forelimb and the poor ossification of the distal limb, this suggests that the forefins were disproportionally small in life. The hind fins appear to have been even smaller than the forefins, and the humerus to femur ratio is 1.16. However, the proximal bones of the hindfins retain a shaft and are remarkably stout (Fig. 4B). The hindfins also appear poorly ossified distally.

### Reevaluation of *Shastasaurus pacificus*

**Figure 5:**
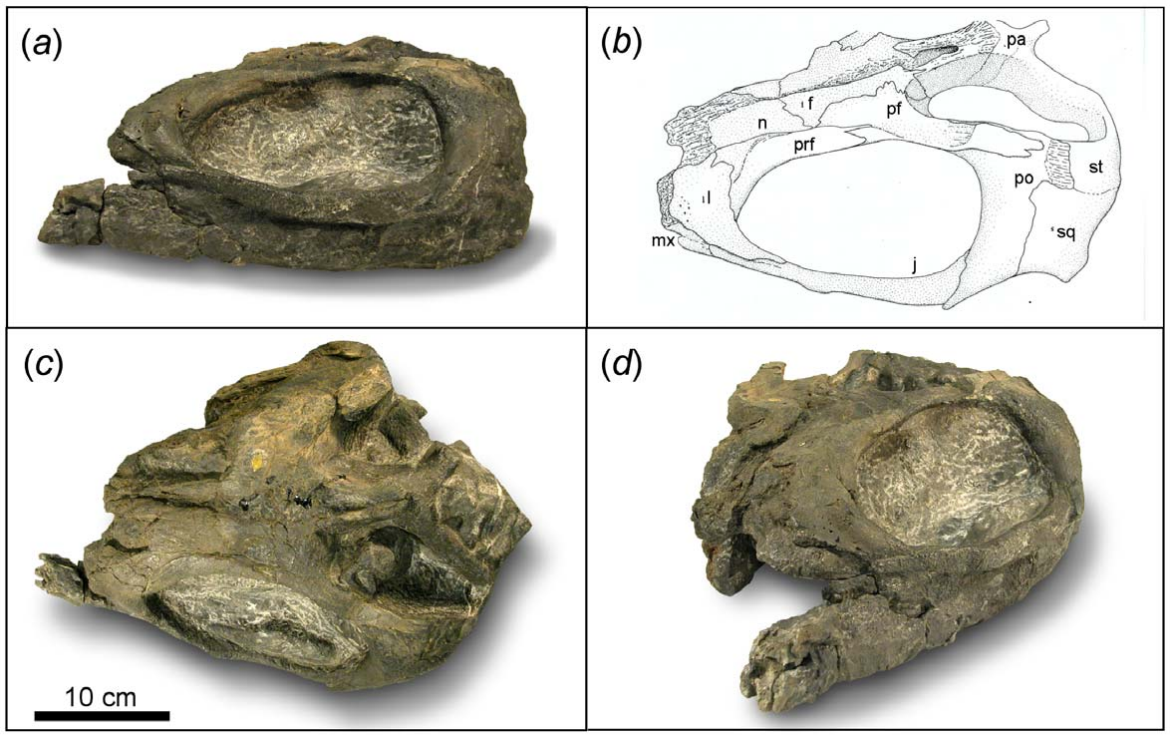
Partial skull of *Shastasaurus pacificus* (UCMP 9017) from the Late Triassic of California, USA, in (A) lateral, (B) dorsal, and (C) anterolateral view. Based on this skull, *Shastasaurus* has repeatedly been reconstructed with a long, tooth-bearing rostrum. However, note the slenderness of the lower jaw (B, C) and the strong anterior taper of the snout (B), both of which are more consistent with the abbreviated and toothless snout of *Shastasaurus liangae* comb. nov. than with the traditional long-snouted reconstruction of this skull (as, e.g., in references [22] and [23]).

*Shastasaurus pacificus* was the first large ichthyosaur from the Triassic for which articulated material became known. It was first described over a century ago based on a specimen from the late Carnian of northern California [15], [22]. Although the rostrum remains unknown, being broken off in the only reasonably complete skull, UCMP 9017 (Fig. 5), the preserved parts of maxilla and dentary in this skull are toothless [23]. Notwithstanding, *Shastasaurus pacificus* was reconstructed several times in the past to conform to the general ichthyosaurian skull shape with a long rostrum and numerous teeth [e.g.], [ 7], [17], [23,24]. These reconstructions were apparently inspired by the putative assignment of a normal ichthyosaurian snout fragment from the Upper Triassic of Mexico to *Shastasaurus altispinus* [5], [20], [24] and on the assignment of a long-snouted ichthyosaur from the Norian of British Columbia to *Shastasaurus* as *S. neoscapularis* [25]. However this material has since been placed in its own genus, *Callawayia* [5], [7], [20]. We follow the view of Nicholls & Manabe [13], [20] that the genus *Shastasaurus* should be restriced to Merriam's [15], [26] original type series from the Hosselkus Limestone of California. The notion of *Shastasaurus* being a typical, long-snouted ichthyosaur is also reflected in the recent reassignment by Shang & Li [19] of *Guizhouichthyosaurus tangae* to the genus *Shastasaurus*.

Re-examination of UCMP 9017 in light of the new Chinese material and character optimization based on phylogenetic analysis and suggests that *S. pacificus* (Fig. 5) had the same reduced snout as *Shastasaurus liangae* comb. nov. In addition, among the material from California, distal limb elements are rare and not preserved in articulation, and isolated teeth were never found with any of the Californian *Shastasaurus* material [15], [22], [26]. These observations are consistent with the edentulous condition and reduced distal fins of *Shastasaurus liangae* comb. nov. However, some anatomical differences, such as the relatively longer postorbital region, the larger upper temporal openings, and the posteriorly diverging parietals indicate that *Shastasaurus liangae* is not a junior synonym of *Shastasaurus pacificus* (see diagnosis).

### Phylogenetic relationships and taxonomic consequences

**Figure 6:**
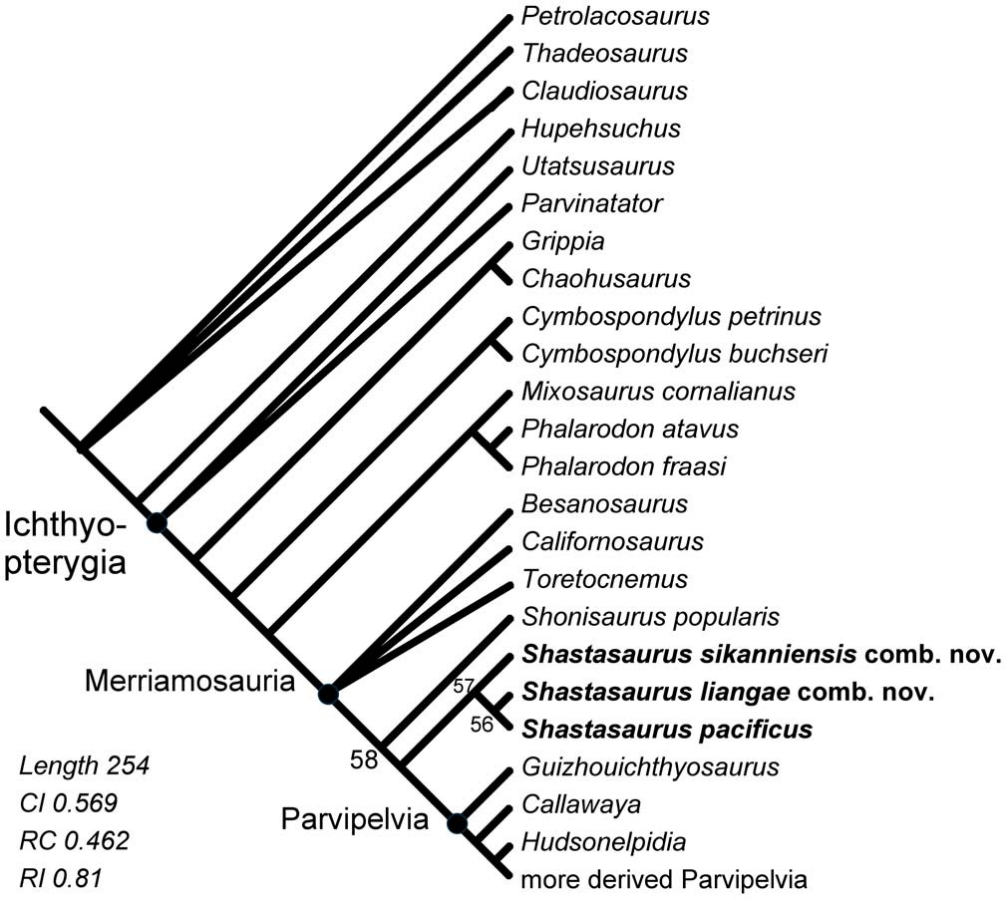
Phylogenetic relationships of *Shastasaurus*. This cladogram represents the strict consensus of 72 most parsimonious trees. Differences in topology among MPTs are mainly found among the outgroup taxa and the basal Merriamosauria. Derived Parvipelvia were part of the analysis but were omitted for clarity. Relevant nodes are numbered in accordance with Table S5. See Materials and Methods section and Supporting Information for details of analysis.

A very similar tree topology resulted from both phylogenetic analyses, one based on a modified and extended data matrix of Motani [17] and one based on a modified and extended data matrix of Sander [6]. In this article, we will discuss the analysis of the modified matrix of Motani [17] in detail because it represents the most widely used data set in ichthyosaur phylogenetic research. Our analysis based on the modified and extended data matrix of Motani [17] recovered 72 most parsimonious trees (MPTs). The strict consensus of these (Fig. 6) had a length of 254 steps, a consistency index of 0.569, a rescaled consistency index of 0.462, and a retention index of 0.81. The disagreement between the MPTs is largely confined to the outgroup taxa and the basal Merriamosauria. Exept for these poorly resolved areas and the new taxa, the topology of the tree does not differ from that found in the earlier analysis by Motani [17]. The quality metrics of our analysis do not differ much from that of Motani [17] either, which had a consistency index of 0.654 and was one step shorter. The sligthly poorer measures for the tree statistics in our analysis are not surprising because of the addition of four terminal taxa and of new characters. However, we note that the scope of the current study is not a reanalysis of ichthyosaurian interrelationships but the determination of the phylogenetic position of *S. liangae*.

In the analysis of the modified matrix of Motani [17], *Shastasaurus liangae* comb. nov. is most closely related to *Shastasaurus pacificus* (Fig. 6). These two taxa in turn are most closely related to *Shonisaurus sikanniensis*, forming a monophyletic group. *Shonisaurus popularis* was found to be less derived than this clade, making the genus *Shonisaurus* paraphyletic. This leads us to propose including *S. sikanniensis* in the genus *Shastasaurus* as *Shastasaurus sikanniensis* comb. nov. We feel justified in doing so because the original authors [13] had already noted the strong affinites of this species with *Shastasaurus*, and their decision to assign *S. sikanniensis* to *Shonisauris* was not based on a phylogenetic analysis.

We used the unambiguous and unequivocal synapomorphies at node 57 (abbreviated rostrum and slender lower jaw, see Table S5) to diagnose *Shastasaurus* because of the major morphological departure from all other ichthyosaurs they represent. Retaining the original genus names for *S. sikanniensis* and *S. liangae* was not an option because of the resulting paraphyly of the genus *Shonisaurus*. Since the genus name *Shonisaurus* has to stay with the type species *S. popularis*, the only taxonomic options for *S. sikanniensis* were either to erect a new genus name or to include it and *G. liangae* in the genus *Shastasaurus*. The list of apomorphies for *Shastasaurus* and the three included species are provided in Table S5.

## Discussion

### Toothlessness in ichthyosaurs

Previously, complete toothlessness had only been described for the adults of one other Triassic ichthyosaur species, i.e., the giant *Shastasaurus sikanniensis* comb. nov. However, the juveniles of the *S. sikanniensis* appear to have had teeth [13]. Additionally, Nicholls & Manabe [13] suggested that *Shonisaurus popularis* also lacked teeth in the adult, but this is difficult to verify because of the poor preservation of the material.

*S. sikanniensis* comb. nov. resembles *S. liangae* comb. nov. in its toothlessness, in that its snout is reduced, the hyoids are enlarged, and at <3 m [13], the skull is small relative to the estimated body length of 21 m. Our phylogenetic analysis (Fig. 6) indicates that the Shastasauridae evolved towards tooth reduction and loss, possibly beginning with *Besanosaurus leptorhynchus* which has few and very small teeth [27]. The short snout of *Shastasaurus liangae* comb. nov. thus may have evolved from a long-snouted ichthyosaur with a slender rostrum like *Besanosaurus* by strong heterochrony. We hypothesize that early developmental stages of this ancestor that were retained by *Shastasaurus liangae* comb. nov. are the failure of teeth to form, the participation of the nasal and angular in the tip of the snout, and the very large internasal foramen. In embryos of extant Reptilia, the jaws ossify well before the development of teeth [28]. Similarly, at least in *Lacerta* and *Sphenodon* embryos, the premaxillary bones are separated among the skull midline by the nasals which reach the tip of the snout [28]. The fairly late-stage embryos of the sauropodomorph dinosaur *Massospondylus* are toothless as well [29], but early hatchlings have a full complement of teeth. The alternative hypothesis to explain the evolution of toothlessness in *Shastasaurus*, early senescence of the dental lamina, would require senescence to have occurred in early juveniles, requiring a much greater shift in development than the first hypothesis.

Although toothlessness occurs in mature individuals of a few Jurassic ichthyosaur species [5], [13], the toothless condition in some of these is a taphonomic artifact because of the loose, aulacodont tooth implantation [5]. None of the Jurassic forms has a smooth dorsal surface of the dentary without a trace of a dental groove or tooth sockets [5], and none has the strikingly reduced snout of *Shastasaurus liangae* comb. nov., which only accounts for 42% of lower jaw length (the snout ratio of McGowan [30]), as compared to 53% in the Jurasssic ichthyosaur with the shortest snout, *Ichthyosaurus breviceps* [30].

### Modern analog

The closest modern analogs in skull shape, body proportions and body size to *Shastasaurus liangae* and the lesser known other two species of *Shastasaurus* are the unusual Ziphiidae (beaked whales, Odontoceti [31], [32]) which range from 6 m to 11 m in length and have a proportionally small skull and a snout that is toothless, save for one or two pairs of peculiar teeth in the lower jaw, which do not erupt in the females of some species. In addition, ziphiids share with *Shastasaurus liangae* an elongate body and very small forefins [31]. As in *Shastasaurus* species, their toothlessness evolved from ancestral forms with numerous teeth [11], [33], [34]. Further similarities in the skull of *Shastasaurus liangae* and ziphiid whales are the dorsally convex coronoid region of the lower jaw and the enlarged hyoids. Other modern odontocetes tending towards toothlessness are the modern sperm whales (*Physeter* and the pygmy and dwarf sperm whales, *Kogia sima* and *K. breviceps*), some delphinids such as Risso's dolphin (*Grampus griseus*, a few lower teeth and no upper teeth), and the narwhal (*Monodon monoceros*) which only retains its huge maxillary tusk [32]. Among these, *Kogia* spp., *Grampus*, and *Monodon* have an abbreviated rostrum as seen in *Shastasaurus*.

These odontocete taxa and the ziphiid whales are suction feeders in which the tongue is pulled back rapidly by strong retractor muscles that are attached to hypertrophied hyoid bones [10]. In this way, prey items are taken up by the negative pressure generated in the oral cavity, obviating the need for teeth to hold them before swallowing. Based on the morphological similarity of suction-feeding odontocete whales and *Shastasaurus liangae* comb. nov., including the enlarged hyoids and the massive convex coronoid region of the lower jaw, and by exclusion of other options, we suggest that *S. liangae* comb. nov., and probably *S. pacificus* and *S. sikanniensis* comb. nov., were specialized in a similar manner (see also Nichols & Manabe [13] on *S. sikanniensis*). An importantant component of the diet of most suction-feeding odontocetes, particulary of ziphiids [31], [35], are coleoid cephalopods. Cephalopods, which are commonly bioluminescent, would have been suitable prey for visually hunting ichthyosaurs [36], [37], [38] as well, especially since bioluminescent cephalopods would have been visually detectable below the photic zone and at night. The eyes of *Shastasaurus* species appear to be average-sized for ichthyosaurs, although this is difficult to quantify because of lack of comparative size standards. They were clearly relatively larger than in *Cymbospondylus* but smaller than in mixosaurs and *Qianichthyosaurus* [5], [6], [39].

Werth [11] established the relationship between an abbreviated snout and generation of suction in modern cetaceans but, as the example of the ziphiids shows, an abbreviated rostrum is not a requirement for suction feeding. The specific mechanism of suction feeding in *Shastasaurus* probably was different from that of ziphiid and other whales, because the latter have a secondary soft palate that aids in generating negative pressure. The exact mechanism of suction generation is difficult to infer in *Shastasaurus* but may have involved specialized soft tissue structures in the snout such as lips or cheeks that would have tightly closed the mouth on the sides and increased the efficency of suction. The potential presence of such structures is hinted at by the unique large foramina in the maxillary and lacimal bones of *S. liangae* comb. nov. The massive retroarticular process of the lower jaw, combined with the reduced rostrum, may also have played a role in suction feeding because they would have allowed very fast and forceful opening of the jaws. Preferred prey of *Shastasaurus* may have been pelagic coleoid cephalopods and fish. Both of these are fast swimmers, making suction feeding a more efficient hunting mechanism than the elongated, tooth-bearing rostrum plesiomorphic for ichthyosaurs in general and Merriamosauria in particular. Shelled cephalopods, predominantly ammonites, are slower swimmers and occur abundantly together with *Shastasaurus* but may have been less attractive prey for *Shastasaurus* because of their protective shell and rounded shape.

As noted, further similarities of *Shastasaurus* and Ziphiidae are the very small pectoral fins and the slender, elongate body. In ziphiids, the reduced pectoral fins are believed to be an adaptation to deep diving, reducing drag on the descent. The functional significance of body elongation and high vertebral number in *Shastasaurus liangae* comb. nov. is not clear. High vertebral numbers are typical of marine reptiles [21], and *S. liangae* comb. nov. appears to be the culmination of this evolutionary trend among ichthyosaurs.

Notably, just like the modern suction-feeding odontocetes [32], the species of *Shastasaurus* show a size range from about 4 m to about 20 m adult body length. This similar size bracket may have biomechanical reasons rooted in the scaling of muscle power and maneuverability. Whales larger than 20 m (exclusively mysticetes) are filter feeders, whereas a body size <4 m, seen in most dolphins and toothed ichthyosaurs, may favor catching prey with a long slender rostrum studded with teeth.

### Diversification of suction feeding ichthyosaurs

Our detailed study of the osteology of the *Shastasaurus liangae* comb. nov. and the phylogenetic analysis of related taxa reveals a Late Triassic diversification of large, toothless, suction-feeding ichthyosaurs. The spatial and temporal distribution of the species of *Shastasaurus* indicates that this diversification was widespread, if not global, and long-lasting, from at least the early Carnian to the middle Norian. Considering the only recently recognized [40] very long duration of the Late Triassic, especially the Norian, the diversification of suction feeding ichthyosaurs thus may have lasted for up to 32 million years and may represent a hidden radiation in the Triassic oceans, only incompletely captured by the fossil record.

Support for this view comes from the small number, low diversity, and limited geographic spread of Late Triassic ichthyosaur localities compared to those of Middle Triassic age [5]–[8], especially considering the vastly longer duration of the Late Triassic compared to the Middle Triassic [40]. Add to this the apparently pelagic lifestyle of *Shastasaurus* and a similar picture emerges as for the ziphiid whale fossil record. Because of their pelagic life style and deep-diving habit, these whales are not only very rare in fossil whale faunas [33], [34], [41] but are also the least known group of whales today. A pelagic lifestyle for *Shastasaurus* is suggested by its rarity in the Xiaowa Formation of China and the Hosselkus Limestone of California and also by the observation that all of the beds that yielded *Shastasaurus* have a pelagic depositional environment as well as a distinctly pelagic fauna of ammonites, halobiid bivalves and vertebrates [13], [16], [22]),

### Suction feeding and low atmospheric oxygen

Restudy of *Shastasaurus liangae* comb. nov. from China and *Shastasaurus pacificus* from the United States adds another, unexpected type to the already very diverse feeding adaptations in Triassic ichthyosaurs [36] and reveals a Late Triassic diversification of large to giant toothless, presumably suction-feeding ichthyosaurs. By the Late Triassic, ichthyosaurs had evolved the widest range of trophic adaptations known in any marine tetrapod group [6], [8], [36], [42]. The diversification of ichthyosaurs in the Early and Middle Triassic happened together with the recovery of marine invertebrate life from the devastating end-Permian extinction [1], [2]. In particular, ammonites apparently recovered much more quickly than the benthic invertebrates [40]. If this is indicative of a fast recovery of cephalopods in general, it would explain the rapid radiation of early ichthyosaurs, because shell-less cephalopods were the main prey of ichthyosaurs. Apart from the diversity of standard ichthyosaurs (cephalopod and fish eaters with an isodont dentition of small conical teeth) and the putative suction feeders described here, the Triassic witnessed the evolution of ichthyosaurs belonging to other feeding guilds [36]. These include forms with crushing dentitions (*Phalarodon* [43], *Tholodus* [44], *Xinminosaurus* [45]) and, in the Norian, a large marine top predator (*Himalayasaurus* [46]). The appearance of *S. liangae* comb. nov. in the poorly oxygenated environment of the Carnian Xiaowa Formation [16] and the diversification of suction-feeding ichthyosaurs is consistent with the Phanerozoic minimum in atmospheric oxygen in the Late Triassic, which may have given air-breathing marine reptiles and low oxygen-tolerant cephalopods a competitive advantage over gill-breathing fish [4], [16].

Suction-feeding ichthyosaurs of the genus *Shastasaurus* did not survive into the Jurassic. This may have been because they lost their competitive advantage as atmospheric oxygen rose again, although there are no other large suction feeders known among Early Jurassic marine vertebrate. Alternatively, suction-feeding ichthyosaurs may have fallen victim to the end-Triassic extinction event that greatly reduced ichthyosaur taxonomic and ecological diversity [6], [42], with only the thunniform longirostrine Neoichthyosauria surviving. These radiated throughout the Jurassic but never reached the trophic diversity of Triassic forms again, including the suction-feeding *Shastasaurus*.

## Supporting Information

Table S1 The additional characters in the modified and extended character matrix from Motani [17].
(DOC)

Table S2 Characters states in the four new terminal taxa in the modified and extended character matrix from Motani [17].
(DOC)

Table S3 Coding of the six new characters for all taxa of the modified and extended character matrix from Motani [17].
(DOC)

Table S4 NEXUS file of the modified and extended character matrix from Motani [17] used in the phylogenetic analysis.
(TXT)

Table S5 List of apomorphies found in the phylogenetic analysis based on the modified matrix from Motani [17]. ACCTRAN = optimization criterion accelerated transformation, DELTRAN = optimization criterion delayed transformation. Bold print indicates unambiguous and unequivocal synapomorphies.
(DOC)
