# Supplementary material for: Short-Snouted Toothless Ichthyosaur from China Suggests Late Triassic Diversification of Suction Feeding Ichthyosaurs
Source: PLoS One. 2011 May 23;6(5):e19480. doi: 10.1371/journal.pone.0019480 (PMC3100301; doi:10.1371/journal.pone.0019480)
Supplement: Table S1 — The additional characters in the modified and extended character matrix from Motani [17]. (DOC) [file pone.0019480.s001.doc]

**Table S1.** The additional characters in the modified and extended character matrix from Motani [17].

106. Maxilla (0) unreduced or moderately reduced (1) strongly reduced but tooth-bearing

(2) reduced, no teeth

1. Abbreviated rostrum (0) absent (1) present
2. Lower jaw (0) normal development (1) slender, reduced in diameter
3. Marginal teeth (0) well developed (1) reduced (2) lost
4. Ischium (0) plate-like (1) stout with rounded shaft

111. Lacrimal with numerous small to medium-sized nutritive foramina (0) absent (1) present
