# Supplementary material for: Short-Snouted Toothless Ichthyosaur from China Suggests Late Triassic Diversification of Suction Feeding Ichthyosaurs
Source: PLoS One. 2011 May 23;6(5):e19480. doi: 10.1371/journal.pone.0019480 (PMC3100301; doi:10.1371/journal.pone.0019480)
Supplement: Table S2 — Characters states in the four new terminal taxa in the modified and extended character matrix from Motani [17]. (DOC) [file pone.0019480.s002.doc]

**Table S2.** Characters states in the four ne terminal taxa in the modified and extended character matrix from Motani [17].

*Guizhouichthyosaurus tangae* 2 1 1 1 1 0 0 1 1 1 1 1 0 1 1 1 1 1 0 1 1 ? 1 1 0 2 1 1 ? ? 1 1 0 1 ? 1 1 0 0 0 0 0 0 1 ? 0 1 1 2 0 1 1 1 1 0 0 0 1 2 2 1 2 2 1 1 1 ? 3 1 1 2 0 0 1 0 0 1 1 1 1 ? 1 1 1 0 0 0 0 1 0 0 0 1 ? 2 1 1 0 1 0 1 0 1 1 1 0 0 0 0 0 0

Sources are personal observations and the studies by Maisch et al. [17] and Shang & Li [18].

*Shastasaurus sikanniensis* comb. nov. ? ? ? ? 1 ? ? ? ? 1 ? ? ? ? ? 0 0 0 0 ? ? ? ? ? 0 2 ? 1 ? ? ? 1 ? 0 ? ? ? ? ? ? ? ? ? 1 1 0 ? 1 ? 0 1 1 1 1 0 0 0 1 2 1 1 2 0 1 ? ? 1 ? ? ? ? ? ? ? ? ? ? ? ? ? ? ? ? ? ? ? ? ? ? ? ? ? ? ? ? ? 1 0 1 0 1 0 ? ? ? 2 1 1 2 ? 0

The source is Nicholls et al. [12] and limited personal observations.

*Shastasaurus pacificus* ? ? ? ? ? 0 0 ? 1 1 1 ? 1 1 ? 1 ? 1 0 1 ? ? 0 1 0 2 1 1 ? 0 1 ? ? ? ? ? ? ? ? ? ? ? ? 1 ? 0 1 ? 2 0 1 1 1 1 0 0 0 1 ? ? 1 2 0 1 1 ? 1 ? 1 ? ? ? ? ? 0 0 ? ? ? 1 ? ? 1 1 0 0 0 0 ? 1 0 0 1 ? ? ? 1 ? 1 0 1 0 ? ? ? ? ? 1 ? 1 1

Sources are personal observations and the studies by Merriam [13,20].

*Shastasaurus* *liangae* comb. nov. 2 0 0 1 1 0 0 1 1 1 0 1 1 1 1 1 0 1 0 1 1 1 0 1 0 ? ? ? ? ? 1 0 0 0 ? ? ? ? ? ? ? ? ? ? ? 0 1 1 2 0 1 1 1 1 0 0 0 1 2 2 1 2 2 1 0 1 ? ? 1 ? ? ? ? 1 ? 0 ? 1 1 1 ? ? 1 ? 0 0 0 0 ? 1 0 0 1 ? 2 ? 1 0 1 0 1 0 ? 1 ? 2 1 1 2 1 1

All character states were scored based on personal observations of the specimens described herein.
