# Supplementary material for: Short-Snouted Toothless Ichthyosaur from China Suggests Late Triassic Diversification of Suction Feeding Ichthyosaurs
Source: PLoS One. 2011 May 23;6(5):e19480. doi: 10.1371/journal.pone.0019480 (PMC3100301; doi:10.1371/journal.pone.0019480)
Supplement: Table S3 — Coding of the six new characters for all taxa of the modified and extended character matrix from Motani [17]. (DOC) [file pone.0019480.s003.doc]

**Table S3.** Coding of the six new characters for all taxa of the modified and extended character matrix from Motani [17].

106 107 108 109 110 111

*Petrolacosaurus* 0 0 0 0 0 0

*Thadeosaurus* 0 0 0 0 0 0

*Claudiosaurus* 0 0 0 0 0 0

*Hovasaurus* 0 0 0 0 0 0

*Hupehsuchus* ? 0 0 2 0 0

*Utatsusaurus* 0 0 0 0 0 0

*Grippia* 0 0 0 0 0 0

*Parvinatator* 0 0 0 0 0 0

*Chaohusaurus* 0 0 0 0 0 0

*Cymbospondylus petrinus* 0 0 0 0 0 0

*Cymbospondylus buchseri* 0 0 0 0 ? 0

*Mixosaurus cornalianus* 0 0 0 0 0 0

*Mixosaurus atavus* 0 0 0 0 ? 0

*Mixosaurus nordenskioeldii* 0 0 0 0 0 0

*Besanosaurus* 0 0 0 1 0 0

*Shonisaurus popularis* 2 0 0 1 0 ?

*Californosaurus* ? ? ? ? 0 ?

*Toretocnemus* 0 0 0 0 0 ?

*Hudsonelpidia* ? 0 0 ? 0 0

*Macgowania* 1 0 0 0 ? 0

*Suevoleviathan* 1 0 0 0 0 0

*Temnodontosaurus* 1 0 0 0 0 0

*Leptonectes* 1 0 0 0 0 0

*Excalibosaurus* 1 0 0 0 ? ?

*Eurhinosaurus* 1 0 0 0 0 0

*Ichthyosaurus* 1 0 0 0 0 0

*Stenopterygius* 1 0 0 0 0 0

*Brachypterygius* 1 0 0 0 0 0

*Ophthalmosaurus* 1 0 0 0 0 0

*Caypullisaurus* 1 0 0 0 0 0

*Platypterygius* 1 0 0 0 0 0

*Guizhouichthyosaurus* 0 0 0 0 0 0

*Shonisaurus sikanniensis* 2 1 1 2 ? 0

*Shastasaurus pacificus* ? ? 1 ? 1 1

*Shastasaurus liangae*  2 1 1 2 1 1

*Callawaya* 1 0 0 0 0 0
