# Supplementary material for: Short-Snouted Toothless Ichthyosaur from China Suggests Late Triassic Diversification of Suction Feeding Ichthyosaurs
Source: PLoS One. 2011 May 23;6(5):e19480. doi: 10.1371/journal.pone.0019480 (PMC3100301; doi:10.1371/journal.pone.0019480)
Supplement: Table S5 — List of apomorphies found in the phylogenetic analysis based on the modified matrix from Motani [17]. ACCTRAN = optimization criterion accelerated transformation, DELTRAN = optimization criterion delayed transformation. Bold print indicates unambiguous and unequivocal synapomorphies. (DOC) [file pone.0019480.s005.doc]

**Table S5.** List of apomorphies found in the phylogenetic analysis based on the modified matrix from Motani [17]. ACCTRAN = optimization criterion accelerated transformation, DELTRAN = optimization criterion delayed transformation. Bold print indicates unambiguous and unequivocal synapomorphies.

Optimization Char. # ci State Change Derived State

node_58 --> node_57 (*Shastasaurus*)

ACCTRAN 23 0.500 1 --> 0 jugal/quadratojugal dorsal contact absent

**107 1.000 0 ==> 1** abbreviated rostrum present

**108 1.000 0 ==> 1** lower jaw slender, reduced in diameter

109 0.500 0 ==> 2 marginal teeth lost

110 1.000 0 --> 1 ischium stout with rounded shaft

DELTRAN 106 0.667 0 --> 2 maxilla reduced, no teeth

**107 1.000 0 ==> 1** abbreviated rostrum present

**108 1.000 0 ==> 1** lower jaw slender, reduced in diameter

109 0.500 0 ==> 2 marginal teeth lost

node_57 --> (*S. sikanniensis*)

ACCTRAN 16 0.333 1 --> 0 sagittal eminence absent

18 0.200 1 ==> 0 parietal supratemporal process short

60 0.500 2 ==> 1 radius contiguous shaft notch or largely reduced

DELTRAN 18 0.200 1 ==> 0 parietal supratemporal process short

60 0.500 2 ==> 1 radius contiguous shaft notch or largely reduced

node_57 --> node_56 (*S. pacificus + S.* *liangae* comb. nov.)

ACCTRAN 32 0.333 1 --> 0 angular lateral exposure extensive

**111 1.000 0 ==> 1** lacrimal with numerous small to medium-sized nutritive foramina

DELTRAN 16 0.333 0 --> 1 sagittal eminence present but small

23 0.500 1 --> 0 jugal/quadratojugal dorsal contact absent

90 0.250 0 --> 1 tibia wider than long

110 1.000 0 --> 1 ischium stout with rounded shaft

**111 1.000 0 ==> 1** lacrimal with numerous small to medium-sized nutritive foramina

node_56 --> *S*. *liangae*  comb. nov.

ACCTRAN 11 0.500 1 ==> 0 postorbital shape triradiate

63 0.333 0 ==> 2 ulna contiguous shaft absent

65 0.167 1 ==> 0 radiale: anterior notch preaxially absent

DELTRAN 11 0.500 1 ==> 0 postorbital shape triradiate

32 0.333 1 --> 0 angular lateral exposure extensive

63 0.333 0 ==> 2 radius contiguous shaft absent

65 0.167 1 ==> 0 ulna contiguous shaft absent

node_56 --> *S. pacificus*

ACCTRAN none

DELTRAN none
